# Reeling in a New Line on Zebrafish Research

**DOI:** 10.3390/biomedicines14010070

**Published:** 2025-12-29

**Authors:** James A. Marrs

**Affiliations:** Department of Biology, Indiana University Indianapolis, 723 West Michigan Street, Indianapolis, IN 46202, USA; jmarrs@iu.edu; Tel.: +1-317-278-0031

## 1. Introduction

In this fourth volume of the *Biomedicines* Special Issue “Zebrafish Models for Development and Disease,” a great variety of advances and scientific syntheses arose from our casting around the vast zebrafish research stream. An exciting set of catches were reeled in! This volume contains papers that examine kidney development, toxicology, models for human disease, retinoic acid signaling, and other topics. The current haul covers new approaches and applications of the zebrafish model that expand its utility and provide new insights, helping it continue to grow and flow.

## 2. Kidney Development

Glycine decarboxylase mutation in human patients produces pleiotropic phenotypes. Rebecca Wingert’s laboratory produced a zebrafish model that recapitulates the disease phenotype, including defects in fluid homeostasis [1]. Expression of the *gldc* gene was strongly detected in the zebrafish embryonic kidney. Gene expression databases showed renal gene expression in humans too. Disrupting *gldc* gene expression in zebrafish reduced renal function. The altered glycine metabolism disrupted renal cell populations in proximal and distal segments, and renal progenitor populations were altered by *gldc* loss-of-function, disrupting renal segment patterning. Loss of *gldc* function increases glycine levels. To test whether the glycine level increase is responsible for the phenotypes, embryos were treated with exogenous glycine, which produced similar renal defects. This study of glycine decarboxylase revealed a mechanism for renal functional defects and specific developmental mechanisms caused by increased glycine levels that directly induce *gldc* loss-of-function phenotypes. This article reflects the growing application of new models to kidney research [2].

## 3. Developmental Toxicity

The zebrafish is gaining greater recognition as a model for testing developmental toxicity [3]. Experiments using zebrafish are much less expensive than rodent models, and greater numbers of individuals are available for analysis. Behavioral assays using the zebrafish model are increasing, making it more powerful. Developmental toxicity using behavior experiments permits correlation of morphological effects on the nervous system with neurobehavioral consequences. The manuscript contributed by da Silva Jr. et al. tested the neurobehavioral effects of essential oils derived from lemongrass, thyme, and oregano [4]. Dietary supplements are gaining popularity and are often used during pregnancy. However, these supplements are not well-regulated, and many of them have not been tested regarding their impact on the safety of the mother and developing child during gestation. The experiments performed by da Silva Jr. et al. [4] illustrate the potential effects of essential oils on morphogenesis and neurodevelopment. These experiments also highlight the need for better testing of dietary supplements in general and particularly for pregnant mothers and developing babies.

## 4. Review and Synthesis

The zebrafish has multiple applications, and the model is expanding into new research arenas [3]. Literature reviews provide summaries, analyses, and syntheses of zebrafish research findings. This Special Issue contains exciting review articles that have illuminated research using the zebrafish for analysis of neurodegenerative disorders and developmental signaling.

Makatbi et al. highlight the growth of the zebrafish model’s use for studying multiple sclerosis [5], helping to advance the zebrafish model’s use for other types of neurological disorders. Multiple sclerosis is a devastating disorder caused by autoimmune attack on the myelin wrappings of neurons in the brain and spinal cord. Developing new models, like those in the zebrafish, opens up new avenues and provides insights into this disorder. The advantages of the zebrafish can be exploited to understand demyelination and potential therapeutic applications. Drug discovery applications in the zebrafish are increasing. Also, the capacity to use the zebrafish for regenerative medicine research will be instructive and could lead to the establishment of remyelination therapies. Makatbi et al.’s article is an excellent review which will boost the zebrafish model’s utilization in neurodegeneration research and therapy development.

Yin and Horzmann provide an extension on the theme of neurodegeneration by reviewing the effects of environmental pollutants and their effects on neurodegenerative disorder etiology [6]. Neurotoxic effects of environmental pollutants produce various forms of damage. These toxins can result in defects related to behavior, cognition, learning, and memory, as well as sensory system defects. Neurodegenerative disorders may also result from toxic insults, which have lasting and potentially devastating effects. The zebrafish offers a platform to evaluate the neurotoxic effects of pollutants. Models used to evaluate specific neurotoxic mechanisms have great value. Engineering the zebrafish model to determine commonalities between different toxic agents will help us better understand how neurodegeneration arises, and these models can be used to compare combinations of insults produced by real-world exposures. The zebrafish model can be developed for use at scale for high-throughput evaluations of compounds. Yin and Horzmann’s review will facilitate the zebrafish model’s growth in the field of neurodegeneration research.

Hawkins and Wingert have contributed an outstanding review of advances made using the zebrafish model to study retinoic acid signaling [7]. Retinoic acid is an important factor that regulates developmental events and adult tissue homeostasis [8,9,10,11]. During development, retinoic acid acts as a morphogen in pattern formation, morphogenesis, and differentiation. Retinoic acid works by transcriptional mechanisms, and the effects of these signals are critical for health and disease. This review explains the zebrafish model’s utility in identifying retinoic acid signaling and biological mechanisms in various developmental and disease syndromes. Overall, this is a comprehensive review that will serve the research community for years to come.

## 5. Conclusions

This Special Issue shows how the zebrafish model is occupying new spaces in biomedical research [12]. In addition to the studies highlighted above, this Special Issue includes other works exploring an array of topics, from the microbiome [13] to skeletal muscle atrophy [14]. New techniques and technologies continue to be applied to the model, yielding insights and advances. Researchers using the zebrafish model will continue to cast their bait to make new insights and novel discoveries, and their research will certainly yield new rewards from the vast ocean of biomedicine.

## Data Availability

Not applicable.
